# Evaluation of a Primary Health Care Scoliosis Screening Program: A 9-Year Follow-Up Study

**DOI:** 10.3390/jcm14113870

**Published:** 2025-05-30

**Authors:** Rafael Rios-de-Moya-Angeler, Fernando Santonja-Medina, Jose Manuel Sanz-Mengibar, Rafael Ríos-Bernabé, José Hurtado-Avilés, Fernando Santonja-Renedo

**Affiliations:** 1 Centro de Salud San Diego, IMIB Pascual Parrilla, Primary Health Service Area III, 30720 Murcia, Spain; rrdemoya@yahoo.es; 2 Faculty of Medicine, University of Murcia, 30100 Murcia, Spain; fernando@santonjatrauma.es (F.S.-M.); joseaviles@um.es (J.H.-A.); 3 Department of Traumatology, V. de la Arrixaca University Hospital, 30120 Murcia, Spain; 4 Centre for Neuromuscular Diseases, National Hospital for Neurology and Neurosurgery, University College London Queen Square, London WC1N 3BG, UK; 5 Primary Health Service Area III, 30720 Murcia, Spain; jenasrios@yahoo.es; 6 Primary Health Service Area IX, 30720 Murcia, Spain; fsr88@hotmail.com

**Keywords:** scoliosis screening, PANA program, scoliometer, adolescent health, primary care

## Abstract

**Background/Objectives:** Scoliosis screening aims to detect spinal deformities early and prevent progression. The Programa de Atención a la Salud de Niños y Adolescentes (PANA) in Spain includes primary care screenings at ages 5–6, 10–11, and 13–14, but its effectiveness remains unverified. First, we evaluated attendance rates in each phase. Second, a nine-year follow-up was used to determine outcomes in adolescents who completed all three phases of PANA. **Methods:** A retrospective–prospective cohort study was conducted. The retrospective phase analyzed records of 881 schoolchildren screened at a primary healthcare center in Lorca, Spain. The prospective phase re-evaluated 127 adolescents (94.1% of those who completed all three phases) after nine years using a standardized forward bending test (FBT) with scoliometer quantification. **Results:** Attendance declined from 73.2%, at age 5–6, to 20.5%, at age 13–14. Only 15.3% completed all three phases. At age 13–14, 11.1% had a positive FBT by visual assessment. Non-quantified FBT had low sensitivity (5.9%) but high specificity (96.7%). Nine years later, mean scoliometer-measured vertebral rotation was 3.6 ± 1.7° (thoracic) and 2.5 ± 1.4° (lumbar). Scoliosis suspicion (FBT > 5°) was 15.1%, but applying the FBT > 7° threshold it was reduced to 4%. **Conclusions:** The PANA program has limited effectiveness due to low attendance and lack of scoliometer use. Visual FBT without quantification increases false positives, reducing diagnostic accuracy. It is recommended that preventive assessments be conducted in schools by primary care physicians. Training in the use of the scoliometer is essential to improve scoliosis detection.

## 1. Introduction

Scoliosis is defined as a “three-dimensional deformity of the spine and trunk” [1]. A commonly used diagnostic criterion is a Cobb angle of 10° or more. Adolescent Idiopathic Scoliosis (AIS) occurs in the general population in a wide range from 0.47% to 5% [2,3].

The relevance of scoliosis screening programs has been widely debated [4,5,6,7], because scoliosis typically occurs without pain during growth; it can typically only be identified through early detection. This approach allows for the diagnosis of curves with lower angular values and helps reduce the number of severe curves identified later, which are more likely to progress [8,9]. For these reasons, several studies positively recommend scoliosis screening programs for their potential benefits in early detection and non-invasive management [10].

Effective detection and timely treatment of scoliosis at disease onset can not only improve AIS patient outcomes but also reduce healthcare costs by avoiding costly surgical interventions, the benefits of which remain controversial [11,12]. Early intervention with bracing has been proven effective in preventing progression to the surgical threshold [13], and rehabilitation has demonstrated efficacy in limiting AIS progression [14].

Although it is universally accepted that the best treatment for scoliosis is achieved when applied in its early stages and that scoliosis often goes undetected unless actively sought, despite this, no consensus has been reached regarding scoliosis screening among various prestigious institutions, such as the Scoliosis Research Society (SRS), the Pediatric Orthopedic Society of North America (POSNA), the American Academy of Pediatrics (AAP), and the American Academy of Orthopedic Surgeons (AAOS) [15]. Screening programs are not recommended by the United States Preventive Services Task Force (USPSTF) [16], the United Kingdom National Health Service (NHS) [17], or the Canadian Task Force on Preventive Health Care (CTFPHC) [18]. The Institute for Clinical Systems Improvement (ICSI) [19] does not provide a clear stance, leaving the decision up to individual responsible authorities.

Among the measurement tools to screen for scoliosis, the scoliometer stands out as a reliable measurement tool [10,20,21] that enhances clinical suspicion precision for scoliosis by quantifying rib prominence in the FBT [22]. Without quantification, FBT may result in a high percentage of false positives (35.5%) [23], underscoring the need for a scoliometer to reduce these high false positive rates.

In Spain, scoliosis screening programs were implemented in schools, following Lonstein’s recommendations. Studies in Murcia assessing all the scholars in the municipality of Murcia indicated a scoliosis prevalence of 1.18% [24], similar to that found by Lonstein in Minnesota, USA (1.2%) [25].

Subsequently, Spanish pediatricians in primary health or general practitioner (GP) surgeons assumed responsibility for spinal assessments under the “Programa de Atención al Niño” (PAN) in Murcia, which includes other aspects of health, such as the vaccination schedule, oral health, BMI, etc. (Supplementary Material Table S1), later extended to the “Programa de Atención al Niño y Adolescente” (PANA) [26,27] implemented in most regions in Spain, including Murcia, Madrid, Castilla-La Mancha, Navarra, Extremadura, Cataluña, Andalucía, and La Rioja. Regarding spinal deformity, this program screens for scoliosis at ages 5, 11, and 14, determining the presence of a “scoliotic morphotype” by assessing posterior trunk alignment and detecting rib prominence during trunk flexion [24,27] in the forward bending test (FBT) [22,28].

The effectiveness of Spain’s scoliosis prevention program (PAN and PANA) remains unexplored, as no studies have been identified that evaluate whether the program has achieved its intended objectives.

This study aims to evaluate the implementation and coverage of the scoliosis screening program within the PANA preventive protocol by analyzing the proportion of children and adolescents assessed across its three phases in a primary care center in Lorca, Spain, during three consecutive years. The secondary objective is to assess the clinical validity (sensitivity and specificity) and safety (positive and negative predictive values) of the screening program.

## 2. Materials and Methods

### 2.1. Study Design

This study utilized a retrospective-prospective design to evaluate the implementation and effectiveness of the PANA scoliosis screening program in primary health care settings. The retrospective phase analyzed clinical records of schoolchildren screened through the program, while the prospective phase involved follow-up assessments nine years later.

#### 2.1.1. Retrospective Study

We conducted a retrospective analysis of medical records from schoolchildren who participated in the PANA program (Programa de Atención a la Salud de Niños y Adolescentes) at the San Diego Health Center in Lorca, Murcia, Spain. Screening assessments were carried out over three consecutive years by the five pediatricians assigned to this health center.

*Data Collection:*

- Attendance records for each of the three mandatory screening phases (ages 5–6, 10–11, and 13–14 years) were reviewed to determine adherence to the program.
- Clinical records were examined to identify findings related to spinal alignment and scoliosis suspicion at each screening phase.

*Screening Protocol*

**Figure 1:**
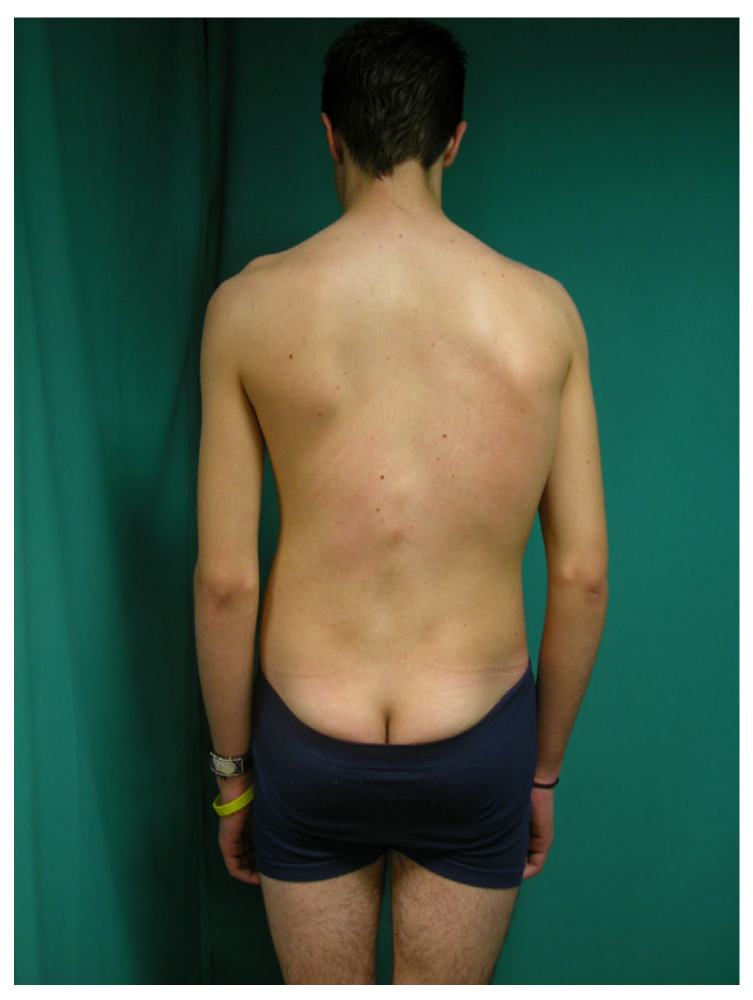
A 14-year-old adolescent with a scoliotic morphotype presenting with right scapular elevation, waist asymmetry, and protrusion of the right scapula and left thoracco lumbar region.

**Figure 2:**
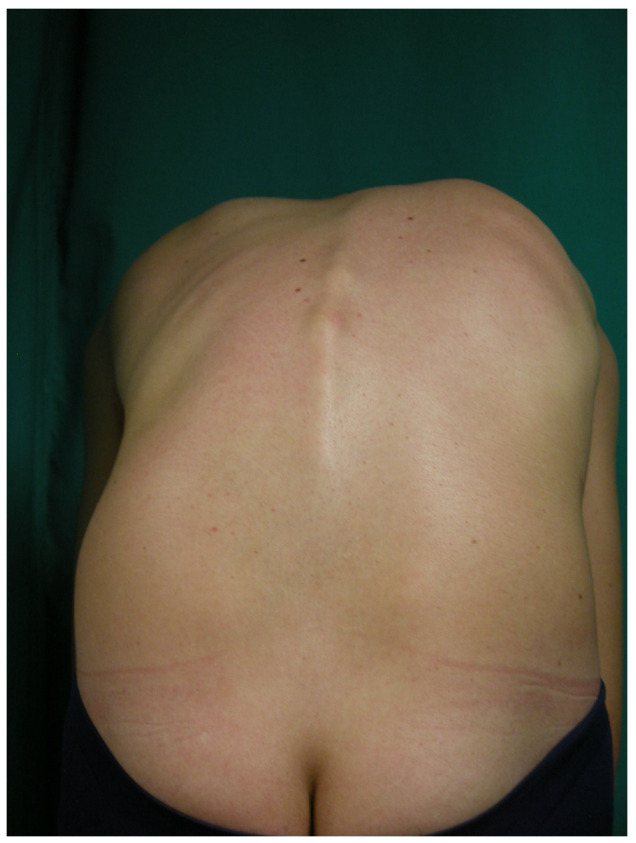
The same 14-years-old adolescent with a positive Forward Benting Test, showing a right thoracic rib prominence.

- The scoliosis screening protocol consisted of a visual back inspection performed without clothing.
- Pediatricians classified children as having a “normal” or “scoliotic morphotype” based on a visual assessment in the standing position, focusing on the presence of asymmetries and/or uneven levels in the back (Figure 1)

- All participants underwent the forward bending test (FBT) to assess for rib prominence (Figure 2). Community pediatricians documented findings as either “normal” or “Adams positive” when asymmetrical rib prominence was detected, suggesting possible scoliosis [24]. However, quantitative measurement was not performed during the initial PANA screenings.

- Children with suspected scoliosis were referred to orthopedic specialists for further evaluation.
- It is important to note that no quantitative measurement of spinal rotation was performed during these screenings, as a scoliometer was not provided as standard equipment by the Servicio Murciano de Salud.

*Study Population and Participants*

**Figure 3:**
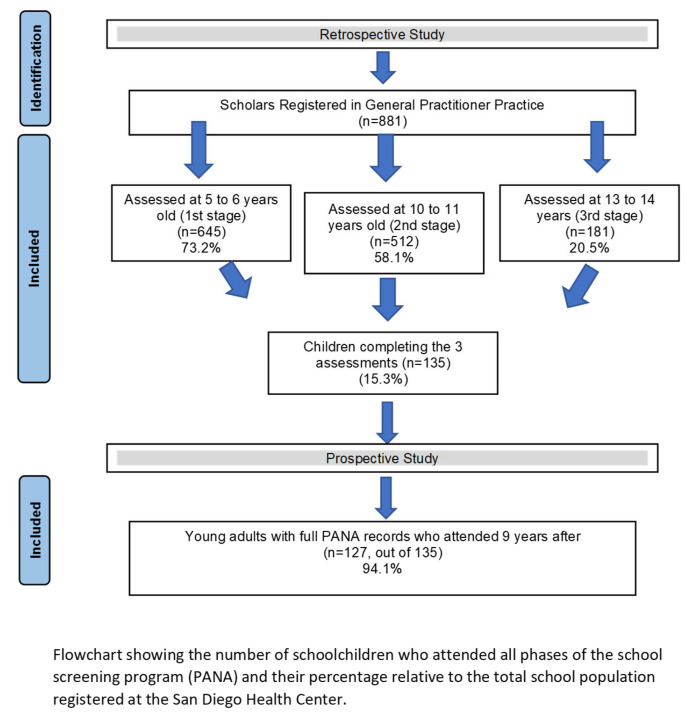
Sample flowchart.

- The initial target population consisted of 881 schoolchildren (both male and female) who were scheduled to participate in all three phases of the PANA health screening program during three years. Figure 3 illustrates the sample distribution and dropout rates across the study timeline.

*Informed Consent:* For the retrospective study, informed consent was granted by the Health Area Manager on behalf of the institution.

Inclusion Criteria: Registered patients at San Diego Health Center in Lorca. Children who were aged 5–6 years at the time of their first scheduled health screening.

Exclusion Criteria: History of spine surgery, history of lower limb surgery that could restrict spinal mobility. Non-attendance across any of the three PANA stages

#### 2.1.2. Prospective Study

Nine years after the completion of the last PANA screening phase, a follow-up prospective assessment was conducted.

*Participant Recruitment*: Adolescents who had completed all three phases of the PANA program (n = 135) were invited via recruitment letters and phone calls to participate in a follow-up spinal assessment.

*Clinical Assessment*

**Figure 4:**
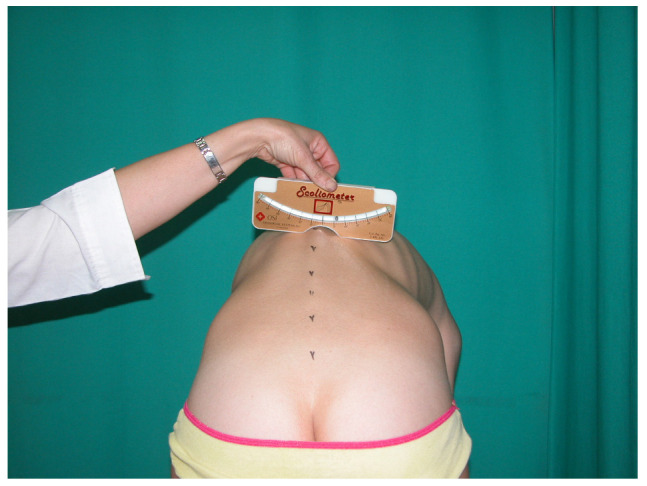
FBT quantifiied with scolimeter, with left rib prominence of 6°.

- A single trained physician conducted the follow-up evaluations to ensure consistency in examination techniques.
- Assessments included a standing posture evaluation and a forward bending test (FBT) with a scoliometer.
- Standing Assessment: Subjects were positioned in neutral posture following standardized protocols [22]. The examiner evaluated for characteristics associated with scoliotic morphotype, including shoulder and scapula height asymmetry, flank and waist triangle symmetry, and rib prominence [22,24].
- Forward bending test with Scoliometer Measurement: During the prospective phase, rib prominences were quantified using a Scoliometer Osi 1995 (Baseline^®^ Scoliometer. Fabrication Enterprises Inc., White Plains, NY, USA) [29] while subjects performed the FBT (Figure 4). In accordance with established clinical guidelines, scoliosis was suspected when vertebral rotation exceeded five degrees [5,8,22].

- The scoliometer used was the Osi 1995 model to quantify vertebral rotation during FBT.

*Examiner Training and Reliability*

- The examining physician underwent specialized training in scoliosis detection techniques.
- Reliability testing was conducted, and the examiner achieved a high intraclass correlation coefficient (ICC > 0.90) for scoliometer measurements, ensuring measurement accuracy and reproducibility.

Inclusion criteria: all adults who complete the three phases of PANA nine years before.

Exclusion criteria: non-attendance across any of the three PANA stages

Statistical analysis

The database was analyzed using SPSS version 25.0 for Windows. Descriptive statistics were obtained through frequency distribution of the variables. Means, standard deviations, and ranges were calculated for quantitative variables, while absolute frequencies were obtained for qualitative variables. Analysis of variance was performed to compare different group samples, and the Student T test was used to assess differences between quantitative and qualitative variables. Simple correlation was calculated between quantitative variables. Tukey’s post hoc multiple comparison test was applied where significant differences were found in ANOVA. Pearson’s correlation coefficient was calculated to examine relationships between scoliometer measurements and visual FBT outcomes. For diagnostic accuracy, sensitivity, specificity, positive predictive value (PPV), and negative predictive value (NPV) were calculated for the FBT to evaluate its diagnostic performance.

## 3. Results

### 3.1. Retrospective Study

The percentage of schoolchildren attending the screening study is low and decreases alarmingly with increasing age. Attendance drops to a concerning 20.5% (181 adolescents out of the 881 who were expected to attend) in the third stage of detection (Figure 1). When analyzing the schoolchildren who completed all three PANA assessments, a total of 135 subjects (15.3%) completed all three assessments of the preventive healthcare program.

FBT results for these 135 subjects were positive in approximately 11% during their initial assessment as schoolchildren, with 12 children aged 10–11 years (9.4%) and 15 adolescents aged 13–14 years (11.8%) testing positive. Trunk alignment observations were not found in the medical records.

### 3.2. Prospective Study

In the subsequent prospective study, 127 schoolchildren (94.1% of the 135 who completed all three assessments of the PANA scoliosis screening) attended the requested follow-up: 75 males (59%) and 52 females (41%). The average age of the final sample was 20.7 ± 1 years.

The quantification of vertebral rotation during FBT in all 127 participants of the prospective study revealed a mean rotation of 3.6 ± 1.7° in the thoracic spine and 2.5 ± 1.4° in the lumbar spine, with both measurements ranging from 1° to 9°. The sample distribution has been summarized in Table 1, and the mean ± SD of the four Adams groups are shown in Figure 2.

Considering 5° as the physiological threshold for vertebral rotation during the FBT [22,29,30], scoliosis suspicion (FBT > 5°) was identified in 15.1% of the subjects, with no significant gender differences (11 males, 14.9%, and 8 females, 15.4%). In the thoracic spine, 18 subjects (14.2%) exhibited an FBT > 5° (Table 1), again with no significant gender differences (10 males, 13.5%, and 8 females, 15.4%). For the lumbar spine, FBT was positive in three males (2.4%), all of whom were males.

**Figure 5:**
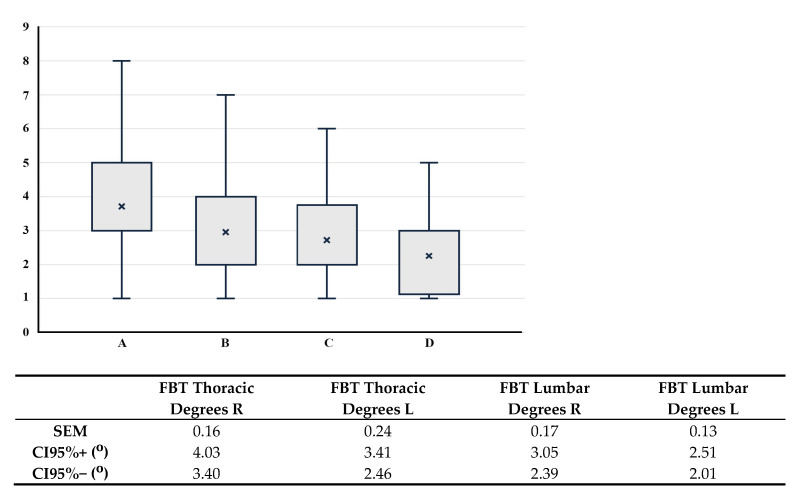
Box-and-whisker plot of measures (in degrees) during FBT at thoracic and lumbar levels in adults after 9 years of the three phases of the screening program, indicating mean, Q1, Q3, and maximum and minimum values. On the vertical axis (ordinate), the distributions of measures in the Adams test are represented (in degrees). On the horizontal axis (abscissa), the four groups are displayed: A = Thoracic Right, B = Thoracic Left, C = Lumbar Right, and D = Lumbar Left. Below, the exact values for the four groups are shown (R = Right; L = Left). SEM = standard error of the mean. CI = confidence interval.

In Figure 5, it can be observed that there are no significant differences among the four groups based on the location of the rib hump (thoracic or lumbar, right or left).

According to the ANOVA analysis, one of these distributions differs from the rest (F = 17.46, *p* < 0.001). Tukey’s multiple comparison test revealed that the variable ‘FBT Thoracic Degrees R’ is statistically significantly different from the other variables in the table (*p* between <0.001 and 0.032), while no statistically significant differences were found among the remaining variables (*p* between 0.101 and 0.908).

When analyzing the diagnostic sensitivity of the pediatricians who conducted the scoliosis screening by correlating subjects with positive results from the subjective, non-quantified FBT during the second and third stages of PANA with the scoliometer values recorded nine years later (Table 2), poor sensitivity was shown (5.9%) and high specificity (96.7%) was observed. Similar specificity (87%) and sensitivity values (5.3%) were found when only considering positive FBT results from the third stage (Table 3).

Table 4 shows the correspondence between the inspection of a scoliotic morphotype and a positive (+) FBT, indicating that the scoliotic morphotype aligns with a positive thoracic FBT in nearly 90% of thoracic Adams tests and in 88% of positive lumbar FBTs.

The values of the FBT measured with a scoliometer in relation to the scoliotic morphotype are presented in Table 5. Complementary information about this table can also be found in Supplementary Material Figure S1.

It was observed that left and right scapular protrusions have thoracic humps of statistically equal size (*p* = 0.233, according to Tukey’s multiple comparison analysis).

The lumbar rib hump shows a statistically significant difference in FBT values (*p* = 0.023) when associated with right scapular protrusion compared to left scapular protrusion. Additionally, the thoracic FBT has a greater angular value than the lumbar FBT (*p* = 0.029).

## 4. Discussion

Our results highlight that the anticipated benefits of schoolchildren visiting health centers for a more comprehensive preventive medical assessment are not being realized. This is in contrast to when the healthcare team (doctor and nurse) conducted assessments at the school, where almost the entire school population was studied, as attendance remains critically low, particularly during puberty, with only 20.5% participation in the prevention program. This leads to missed opportunities for treating scoliosis and other conditions covered by these screening programs. We found no studies investigating attendance rates to PANA screening program, suggesting that it has been assumed that almost the entire target population participates in these evaluations.

The effective screening relies on high attendance. The spine assessment embedded within the overall health programs, like our PANA, could only be effective if there is a high attendance rate at primary care check-ups. In our study, only 15.3% of the target population attended the three recommended follow-ups. When the PANA (School and Adolescent Health Programme) was designed, it was expected to improve detection rates compared to the previous system, where a physician visited schools to assess all students. However, our findings highlight the decline in attendance at the PANA screenings as children grow older, potentially due to reduced parental adherence to preventive healthcare visits, including routine check-ups.

A possible solution could be to emphasize the importance of attending these examinations by sending appointment reminders. The option with the highest likelihood of reaching the vast majority of the population that should benefit from these prevention programs would be to reintroduce screening assessments in schools, conducted by healthcare professionals of the respective health center.

Our second aim was to assess the progression of all adolescents who attended all three check-ups (presumably including especially those who were suspected of having possible scoliosis in the previous evaluations), who were re-evaluated nine years later. The analysis of this population was challenging due to the time elapsed and the high rate of residential relocation. We consider that our systematic and sequential methodology enhanced the results by employing both cross-sectional and longitudinal analyses, providing complementary insights into the subject. A strength of our research in scoliosis screening is that 94.1% of the adolescents who underwent the full PANA protocol (assessed at ages 5–6, 10–11, and 13–14) were re-evaluated nine years after their initial check-up.

In our prospective study, a comprehensive clinical examination was performed, and a clinical suspicion of scoliosis was identified in 15.1% of cases (FBT > 5°), which aligns with findings from other national (16% in Granada, Spain) [31] and international studies (17% in Los Angeles, California) [32]. Slightly lower values were found in Bosnia–Herzegovina (11.8%) [33] and Nigeria (7.3%) [34].

If the normative range of the FBT is considered until 7° according to Bunnell, scoliosis suspicion in our sample would have dropped to 3.1% [35]. This percentage is consistent with the generally accepted prevalence of scoliosis (2–4%) in adolescents aged 10–16 years, with studies reporting similar values: Duruwalla at 3.1% [36], Navarro at 3.47% [37], Lonstein at 1.1% [25], Hernández et al. at 1.8% [24], Komang-Agung at 2.93% [38], and Zheng at 2.4% [21]. Differences in percentage of scoliosis suspicion across studies likely reflect variations in assessment methods, such as FBT with scoliometer quantification [21,39,40,41,42], FBT without scoliometer quantification [25,28,31,37], or radiographic studies [24,43].

Recently, Chen et al. (2024) [44] applied ROC curves (receiver operating characteristic) and identified shoulder height difference, scapular tilt, flat back, rib hump (components of a scoliotic morphotype), and thoracic rotation angle as predictors of scoliosis magnitude. These indicators, alongside sex and age, support the utility of inspection in identifying scoliotic morphotypes, which may be valuable for scoliosis screening. This aligns with our observation that the FBT matches the diagnostic suspicion of the scoliotic morphotype in nearly 90% of our population (Table 4).

A systematic review by Dunn et al. (2018) [45] indicated that the accuracy of scoliosis detection increases with the number of screening tools used. Specifically, the FBT with scoliometer demonstrated a sensitivity of 71.1%, specificity of 97.1%, 2.9% false positives, and 28.9% false negatives. Diagnostic accuracy studies for the FBT based solely on visual inspection, where it is determined as positive or negative, have not been identified. Our study demonstrates that sensitivity is very low (around 5%) when the diagnostic suspicion of scoliosis is based solely on inspection during the FBT, without quantifying the degree of the rib hump.

The main objections to early scoliosis diagnosis programs include [46]: (a) high false-positive rates leading to unnecessary radiographic follow-ups, (b) lack of reliable criteria to predict curve progression, (c) limited comparative evidence supporting early intervention benefits, (d) high rates of unnecessary consultations, (e) lack of morbidity studies in untreated scoliosis, and (f) high associated costs. Excessive false positives and non-indicated diagnostic tools can lead to unnecessary interventions; however, sections a, d, and f can be significantly reduced/improved with appropriate training for pediatricians and using a scoliometer, enhancing their diagnostic sensitivity and specificity 15]. Table 3 highlights the limited value of the FBT when performed by untrained physicians and without specific instruments.

The relevance of early diagnosis is the application of conservative treatment [5,47,48,49]. It is accepted that an early diagnosis allows for the initiation of conservative treatment (bracing), preventing the need for surgery and its potential complications [13,50,51].

In 2010, the Scoliosis Research Society (SRS) established an International Task Force to achieve a scientific consensus on scoliosis screening across the US, Canada, Europe, and Asia. Using the Delphi method, the following conclusions were reached [10]: (a) Screening programs are technically, clinically, and therapeutically effective, though cost-efficiency evidence is lacking; (b) The primary aim of screening is early detection, with referrals for confirmation (Cobb Angle > 10° on X-ray). Females should be screened twice between ages 10 and 12, while males should be screened once at 13 or 14 years; (c) The scoliometer is currently the best screening tool, with moderate evidence suggesting that vertebral rotation between 5° and 7° warrants referral. (d) There is moderate evidence that screening enables early detection and referral for idiopathic scoliosis (younger ages and lower curve magnitudes); (e) Early-diagnosed patients are less likely to require surgery; (f) The prevalence, referral rates, and positive predictive values of idiopathic scoliosis support adequate screening; (g) Further improvements in protocols, referral thresholds, and positive predictive values are needed.

In contrast, the PrevInfad group [52] advised that general screening for idiopathic scoliosis has more downsides than benefits and does not recommend systematic detection. Nevertheless, without these programs, many subjects with scoliosis may miss the chance for conservative treatment, potentially leading to severe trunk deformities in adolescence.

If prevention is valued, the key question becomes how to best implement it, minimizing costs while maximizing the detection of students with progressive scoliosis. Bras and Prats (2008) [53] suggested conducting the FBT in children from age 10 during GP visits. We recommend that pediatricians receive training in identifying scoliotic morphotypes to avoid mistaking common thoracic asymmetries (present in 80% of the population) for true scoliosis, as noted by Vercauteren [54] FBT, without quantifying the rib humps, alone increases error rates. Therefore, it is essential to use a scoliometer and ensure that primary care physicians are properly trained in its correct application [22].

Álvarez y Núñez [5] proposed the following primary care protocol: scoliosis can be ruled out if the FBT measures below 5°. For measurements between 6° and 9°, females should undergo six-monthly clinical evaluation follow-ups up to one year post-menarche without radiographic studies. Anterior-posterior and lateral X-rays and specialist referrals are advised if FBT measures 10° or more. Hernández et al. [24] and Santonja et al. [22] suggested that Adams’ test between 5° and 7° with a scoliotic morphotype warrants frontal arrow measurements, and radiographic studies should follow if these are equal to or larger than 10 mm. This avoids missing cases with FBT between 5° and 7°, where scoliosis may still be present, as well as prescribing unnecessary X-rays for cases with FBT between 6° and 7° without scoliosis.

Our study protocol, conducted 9 years after the last PANA screening, included spinal morphotype assessment in a standing position [3,22] and FBT quantification with the scoliometer. A rib hump up to 5° was considered normal [29], though Bunnell suggests a threshold of up to 7°. The FBT showed 92% sensitivity (CI 95% 85–100) for curves with a Cobb angle of 20° on X-ray, though its specificity was lower at 60% (CI 95% 47–74) [37]. This supports the idea that reducing the high proportion of false diagnoses can be achieved by quantifying the Adams test using a scoliometer.

It is important to improve the diagnostic precision of scoliosis to avoid unnecessary X-rays due to false positives. Luan et al. (2020) [55], in a systematic review and meta-analysis of 18,873 scoliosis patients, showed that they underwent an average of 23 X-rays over time (range 0–618) with an estimated radiation dose of 11.35 cGy. Idiopathic scoliosis patients showed an increased cancer incidence ((OR) = 1.46; *p* < 0.00001), including higher breast cancer rates (OR = 1.20; *p* = 0.02) and overall cancer mortality (OR = 1.50; *p* < 0.00001) [55]. This confirms the need to use clinical methods that improve the diagnostic accuracy of scoliosis to reduce false positives, as well as strategies in clinical evaluations to decrease the number of radiographs.

Until it is definitively determined whether scoliosis prevention programs are effective and health systems include programs for scoliosis screening among other diverse conditions, it is crucial to monitor compliance levels and achieve attendance close to 100% of the target population.

Unfortunately, PANA’s shortcomings were not identified in our health program, nor were corrective strategies developed. Detection program efficiency at primary care will only improve if attendance rates increase significantly as close as possible to 100%. We believe that to ensure this, the healthcare team from the GP practice should visit the schools and conduct the evaluations on-site. This approach would allow for the coverage of nearly all scholars, similar to the vaccination programs that, for more than 10 years, were once again moved from the health center back to schools.

Regarding the scoliosis program, changes in screening programs based on subjective FBT are recommended, including scoliometer quantification of the FBT with the scoliometer. Specific assessment training for general practitioners is also required to improve diagnostic accuracy and reduce the percentage of false positives, decreasing the number of full-spine X-rays and unnecessary referrals to musculoskeletal specialist consultations. Implementing these changes could lead to more consistent screening results and better identification of cases that warrant further specialist evaluation.

There were several potential limitations in this study. First, we only have a retrospective cohort study based on the database of a single center of patients originating from a determined region of our country, although we have extended it to three consecutive years to reduce occasional biases, which limits the generalizability of the results to a larger or broader population. Second, it would have been of interest to assess the diagnostic sensitivity of the five pediatricians who conducted the screening evaluations. However, reassessing diagnostic skills is not a common practice in our healthcare system. And third, it would have been desirable to conduct the diagnostic accuracy study with full standing spinal radiographs, but these had not been requested for any of the schoolchildren in our study population in our surgery, and this study reflects the reality of the clinical practice that is performed on them.

In summary, this study highlights significant limitations in the current scoliosis screening program within primary care. Attendance rates in the PANA program were critically low, with only 15.3% of the target population completing all three screening phases and participation decreasing to 20.5% in the final stage (ages 13–14). The expected benefits of conducting screenings in health centers were not achieved. Additionally, the study confirms that relying solely on the forward bending test (FBT) without quantification leads to a high false positive rate, with a diagnostic sensitivity of only 5.9% and a specificity of 96.7%. The use of a scoliometer is essential to enhance diagnostic accuracy, reduce unnecessary referrals and X-rays, and improve overall screening effectiveness.

## 5. Conclusions

Our scoliosis detection system has failed, as only 15.3% of the scholars who were required to attend for “back assessment” for scoliosis screening throughout their growth completed the assessment in our program by attending all three scheduled follow-ups at ages 5–6, 10–11, and 13–14 years within the PANA, which is a comprehensive childhood and adolescence health program. We believe that for the screening program to be effective, future initiatives should consider reminders to attend PANA check-ups or reintroducing school-based screenings, ensuring higher participation rates and early detection of scoliosis.

Quantifying the FBT with a scoliometer is necessary for pediatricians working in primary health care to improve the diagnostic sensitivity of scoliosis screening, and reduce unnecessary referrals and X-rays, and improve overall screening effectiveness.

## Figures and Tables

**Table 1:** Scoliometer quantification of Adam’s test in adults, after 9 years after completing all the three phases of the screening program. Sample distribution.

Interventions/Examinations			Age	
Rotation Degrees	Thoracic Spine		Lumbar Spine	
0–3°	37	29.1 (%)	83	65.4 (%)
4–5°	72	56.7 (%)	41	32.3 (%)
* 6–7°	15	11.8 (%)	1	0.8 (%)
* >7°	3	2.4 (%)	2	1.6 (%)
Total	127		127	

* Double thoracic and lumbar rotation 6–7° was observed in one subject. Double curve > 7° was also described in another subject.

**Table 2:** Relation between scoliosis suspicion with positive subjective forward bending test at age 10 years (second stage) and 14 years (third stage) and the quantification nine years after of the Adam’s test with a scoliometer. Scholars with only a positive in Adam’s test on one of the stages were excluded.

	Scoliometer ≤ 5°	Scoliometer > 5°	Total	
Positive Adam’s test	3 False positives	1 True positives	4	Specificity 96.7% PPV 25%
Negative Adam’s test (Second and third stages)	88 True negatives	16 False negatives	104	Sensibility 5.9% NPV 84.6%
Total	91	17	108	

PPV = positive predictive value; NPV = negative predictive value.

**Table 3:** Relation between scoliosis suspicion with positive subjective Adam’s test at age 10 years (second stage) or 14 years (third stage) and the quantification and validation nine years after of the Adam’s test with a scoliometer.

Forward Bending Test		Scoliometer ≤ 5°	Scoliometer > 5°	Total	
2nd Stage	3rd Stage				
Positive	Negative	7	1	8	
Negative	Positive	11	0	11	
		Specificity	Sensibility	NPV	PPV
Positive	Negative	90.7%	10.5%	85.2%	16.7%
Negative	Positive	87%	5.3%	83.9%	6.7%

PPV = positive predictive value; NPV= negative predictive value.

**Table 4:** Number of cases in which the FBT for the thoracic and lumbar regions does not match with the scoliotic morphotype. The angular value is presented as (X ± SD).

Thoracic FBT		Lumbar FBT	
n	13	n	15
%	10.3	%	11.9
Mean degrees	2.2	Mean degrees	2.3
SD degrees	0.8	SD degrees	0.9

**Table 5:** Relationship between a positive FBT (thoracic and lumbar) and the presence of a scoliotic morphotype with right or left scapular protraction (Scap Prot), as well as a normal back (No Scap Protraction). n = number of cases; mean = mean in degrees; SD = standard deviation; SEM = standard error of the mean; CI = confidence interval.

	Thoracic FBT			Lumbar FBT		
	Scap Prot R	Scap Prot L	No Scap Prot	Scap Prot R	Scap Prot L	No Scap Prot
n	45	62	12	38	63	11
mean (°)	3.78	3.13	3.58	1.71	2.48	2.36
SD (°)	1.80	1.02	1.78	0.61	0.98	0.67
SEM (°)	0.27	0.13	0.51	0.10	0.12	0.20
IC95+ (°)	4.30	3.38	4.59	1.90	2.72	2.76
IC95− (°)	3.25	2.88	2.58	1.52	2.23	1.97

## Data Availability

Data are contained within the article and can be detailed upon request.

## Supplementary Materials

The following supporting information can be downloaded at https://www.mdpi.com/article/10.3390/jcm14113870/s1: Figure S1: 95% Confidence Intervals (Using Z = 1.96)—Updated Data; Table S1: Schedule of Interventions for Routine Child Health Checkups (PANA).

## References

[1] Grivas T.B., Vsiliadis E.S., Rodopoulos G. (2008). Aetiology of idiopathic scoliosis. What have we learned from school screening?. Stud. Health Technol. Inform..

[2] Konieczny M.R., Senyurt H., Krauspe R. (2013). Epidemiology of adolescent idiopathic scoliosis. J. Child. Orthop..

[3] Cheng J.C., Castelein R.M., Chu W.C., Danielsson A.J., Dobbs M.B., Grivas T.B., Gurnett C.A., Luk K.D., Moreau A., Newton P.O. (2015). Adolescent Idiopathic Scoliosis. Nat. Rev. Dis. Primers.

[4] US Preventive Services Task Force (1993). Screening for adolescent idiopathic scoliosis: Policy statement. JAMA.

[5] Álvarez L.I., Nuñez A. (2011). Escoliosis idiopática. Rev. Pediatr. Aten. Primaria.

[6] Plaszewski M., Bettany-Saltikov J. (2014). Are current scoliosis school screening recommendations evidence-based and up to date? A best evidence synthesis umbrella review. Eur. Spine J..

[7] Li X.K., Wu Z.G., Wang H.Q. (2016). Adolescent Idiopathic Scoliosis in China: An Ongoing Warm Debate from Bedside to Public. Spine.

[8] Altaf F., Gibson A., Dannawi Z., Noordeen H. (2013). Adolescent idiopathic scoliosis. Bone Jt. Surg..

[9] Pesenti S., Jouve J.L., Morin C., Wolff S., De Gauzy J.S., Chalopin A., Ibnoulkhatib A., Polirsztok E., Walter A., Schuller S. (2015). Evolution of adolescent idiopathic scoliosis: Results of a multicenter study at 20 years’ follow-up. Orthop. Traumatol. Surg. Res..

[10] Labelle H., Richards S.B., De Kleuver M., Grivas T.B., Luk K.D.K., Wong H.K., Thometz J., Beauséjour M., Turgeon I., Fong D.Y.T. (2013). Screening for adolescent idiopathic scoliosis: An information statement by the scoliosis research society international task force. Scoliosis.

[11] Workman J.K., Wilkes J., Presson A.P., Xu Y., Heflin J.A., Smith J.T. (2018). Variation in Adolescent Idiopathic Scoliosis Surgery: Implications for Improving Healthcare Value. J. Pediatr..

[12] Weinstein S.L., Dolan L.A., Wright J.G., Dobbs M.B. (2013). Effects of bracing in adolescents with idiopathic scoliosis. N. Eng. J. Med..

[13] Negrini S., Donzelli S., Aulisa A.G., Czaprowski D., Schreiber S., De Mauroy J.C., Diers H., Grivas T.B., Knott P., Kotwicki T. (2018). 2016 SOSORT guidelines: Orthopaedic and rehabilitation treatment of idiopathic scoliosis during growth. Scoliosis Spinal Disord..

[14] Kluszczyński M., Zaborowska-Sapeta K., Kowalski I., Karpiel I. (2024). The Effectiveness of Early Rehabilitation in Limiting the Progression of Idiopathic Scoliosis. J. Clin. Med..

[15] Richards S., Vitale M.G. (2008). Screening in idiopathic Scoliosis in Adolescents. An information statement. J. Bone Jt. Surg. Am..

[16] Grossman D.C., Curry S.J., Owens D.K., Barry M.J., Davidson K.W., Doubeni C.A., Epling J.W., Kemper A.R., Krist A.H., Kurth A.E. (2018). Screening for Adolescent Idiopathic Scoliosis: US Preventive Services Task Force Recommendation Statement. US Preventive Services Task Force. JAMA.

[17] Screening for Adolescent Idiopathic Scoliosis: Position Statement. En: NHS. https://posna.org/POSNA/media/Documents/Position%20Statements/1122-Screening-for-the-Early-Detection-of-Idiopathic-Scoliosis-in-Adolescents.pdf.

[18] Goldbloom R. Screening for Adolescent Idiopathic Scoliosis. En: Canadian Services Task Force. http://canadiantaskforce.ca/wp-content/uploads/2013/03/Chapter31_idio_adoles_scoliosis94.pdf?d2b9b5.

[19] Wilkinson J., Bass C., Diem S., Gravley A., Harvey L., Maciosek M., McKeon K., Milteer L., Owens J., Rothe P. (2013). Health Care Guideline. Preventive Services for Children and Adolescents.

[20] Trobisch P., Suess O., Schwab F. (2011). Idiopathic scoliosis. Dtsch. Arztebl. Int..

[21] Zheng Y., Dang Y., Wu X., Yang Y., Reinhardt J., He C., Wong M. (2017). Epidemiological study of adolescent idiopathic scoliosis in Eastern China. J. Rehabil. Med..

[22] Santonja F., Andújar P., Santonja F. (2022). Escoliosis. Manual de Exploración Musculoesquelética.

[23] Yufra D.H., Giordana G. (2011). Escoliosis Idiopática del Adolescente en la Provincia de Jujuy. Chequeo Selectivo 2007–2009. Rev. Asoc. Argent. Ortop. Traumatol..

[24] Hernández J.A., Santonja F., García I., Ortiz E. (1988). Prevalencia de la escoliosis idiopática en Murcia. Rev. Ortop. Traumatol..

[25] Lonstein J.E., Bjorklund S., Wanninger M.H., Nelson R.P. (1982). Voluntary school screening for scoliosis in Minnesota. J. Bone Jt. Surg..

[26] Espín Ríos M., Cervantes Pardo A. (2007). Programa de Atención al Niño y al Adolescente: Guía de Apoyo al Programa.

[27] Navarro Alonso J.A. (1992). Programa de Atención al Niño (PAN). Región de Murcia.

[28] Côté P., Kreitz B.G., Cassidy J.D., Dzus A.K., Martel J. (1998). A study of the diagnostic accuracy and reliability of the scoliometer and Adam’s forward bend test. Spine.

[29] Mittal R.L., Aggerwal R., Sarwal A.K. (1987). School screening for scoliosiss in India. The evaluation of a scoliometer. Int. Orthop..

[30] Lonstein J., John E. (1997). Point of View: Cut-off Point of the Scoliometer in School Scoliosis Screening. Spine.

[31] Zurita Ortega F., Moreno Lorenzo C., Martínez Martínez A., Zurita Ortega A., Castro Sánchez A.M. (2008). Cribado de la escoliosis en una población escolar de 8 a 12 años de la provincia de Granada. An. Pediatr..

[32] Brooks H.L., Azen S.P., Gerberg E.L. (1975). Scoliosis a prospective epidemiologycal study. J. Bone Jt. Surg..

[33] Ostojic Z., Kristo T., Petrovic P., Vasilj I., Santic Z. (2006). Prevalence of scoliosis in school-children from Mostar, Bosnia and Her-zegovina. Coll. Antropol..

[34] Jenyo M.S., Asekun-Olarinmoye E.O. (2005). Prevalence of scoliosis in secondary school children in Osogbo, Osun State, Nigeria. Afr. J. Med. Sci..

[35] Bunnell W.P. (2005). Selective screening for scoliosis. Clin. Orthop. Relat. Res..

[36] Daruwalla J.S., Balasubramaniam P., Chay S.O., Rajan U., Lee H.P. (1985). Idiopathic scoliosis. Prevalence and ethnic distribution in Singapore schoolchildren. J. Bone Jt. Surg..

[37] Navarro J.A., Santonja F., Martínrz I. (1992). Resultados de reconocimiento médico con el programa de salud escolar de la Región de Murcia. Valoración Médico-Deportiva del Escolar.

[38] Komang-Agung I.S., Dwi-Purnomo S.B., Susilowati A. (2017). Prevalence Rate of Adolescent Idiopathic Scoliosis: Results of School-based Screening in Surabaya, Indonesia Malays. Orthop. J..

[39] Weigert K.P. (2006). Detection pattern and outcome assessment in adolescent idiopathic scoliosis. Dan. Med. Bull..

[40] Yong F., Wong H.K., Chow K.Y. (2009). Prevalence of adolescent idiopathic scoliosis among female school children in Singapore. Ann. Acad. Med..

[41] Chen C., Yu R., Xu W., Li Z., Li Y., Hu R., Zhu X. (2020). A Practical Study of Diagnostic Accuracy: Scoliosis Screenings of Middle School Students by a Trained Nurse with a Smartphone Versus a Spine Surgeon with a Scoliometer. Spine.

[42] Cárcamo M., Cárcamo M., Espinoza P., Rodas M., Urrejola Ó., Bettany-Saltikov J., Grivas T.B. (2023). Prevalencia, riesgo de progresión y calidad de vida en estudiantes tamizados para escoliosis idiopática adolescente. Andes pediatr.

[43] Malfair D., Flemming A.K., Dvorak M.F., Munk P.L., Vertinsky A.T., Heran M.K. (2010). Radiographic evaluation of scoliosis: Review. Am. J. Roentgenol..

[44] Chen X., Ye Y., Zhu Z., Zhang R., Wang W., Wu M., Lu X., Yan B., Liang Q. (2024). Association between incorrect postures and curve magnitude of adolescent idiopathic scoliosis in China. J. Orthop. Surg. Res..

[45] Dunn J., Henrikson N.B., Morrison C.C., Blasi P.R., Nguyen M., Lin J.S. (2018). Screening for adolescent idiopathic scoliosis: A systematic evidence review for the U.S. Preventive Services Task Force. JAMA.

[46] British Orthopaedic Association and the British Scoliosis Society (1983). School screening for scoliosis. Br. J. Med..

[47] Gammon S.R., Mehlman C.T., Chan W., Heifetz J., Durrett G., Wall E.J. (2010). A Comparison of Thoracolumbosacral Orthoses and SpineCor Treatment of Adolescent Idiopathic Scoliosis Patients Using the Scoliosis Research Society Standardized Criteria. J. Pediatr. Orthop..

[48] Burton M.S. (2013). Diagnosis and treatment of adolescent idiopathic scoliosis. Pediatr. Ann..

[49] Ceballos-Laita L., Carrasco-Uribarren A., Cabanillas-Barea S., Pérez-Guillén S., Pardos-Aguilella P., Jiménez Del Barrio S. (2023). The effectiveness of Schroth method in Cobb angle, quality of life and trunk rotation angle in adolescent idiopathic scoliosis: A systematic review and meta-analysis. Eur. J. Phys. Rehabil. Med..

[50] Hresko M.T., Talwalkar V., Schwend R. (2016). Early Detection of Idiopathic Scoliosis in Adolescents. J. Bone Jt. Surg. Am..

[51] Altaf F., Drinkwater J., Phan K., Cree A.K. (2017). Systematic Review of School Scoliosis Screening. Spine Deform..

[52] Sanabria A.J., Rigau D., Rotaeche R., Selva A., Marzo-Castillejo M., Alonso-Coello P. (2015). Sistema GRADE: Metodología para la realización de recomendaciones para la práctica clínica. Aten. Primaria.

[53] Bras J., Prats R., Martín A., Cano J.F. (2008). Actividades de prevención y promoción de la salud en la infancia y la adolescencia. Atención Primaria Conceptos, Organización y Práctica Clínica.

[54] Vercauteren M., Van Beneden M., Verplaetse R., Croene P.H., Uyttendaele D., Verdonk R. (1982). Trunk Asymmetries in a Belgian School Population. Spine.

[55] Luan F.J., Wan Y., Mak K.C., Ma C.J., Wang H.Q. (2020). Cancer and mortality risks of patients with scoliosis from radiation exposure: A systematic review and meta-analysis. Eur. Spine J..

