# Supplementary material for: Evaluation of a Primary Health Care Scoliosis Screening Program: A 9-Year Follow-Up Study
Source: J Clin Med. 2025 May 30;14(11):3870. doi: 10.3390/jcm14113870 (PMC12156459; doi:10.3390/jcm14113870)

Supplementary Table S1. Schedule of Interventions for Routine Child Health Checkups (PANA)

Interventions / Examinations	Age		
	5-6 Year	10-11 Years	13-14 Years
Musculoskeletal system (Detection of scoliosis)	X	X	X
Immunizations	X	X	X
Cardiorespiratory system	X	X	X
Blood pressure	X	X	X
Dental examination	X	X	X
Abdomen examination	X	X	X
Somatometry	X	X	X
Auditory system	X	X	X
Visual system	X	X	X
Sexual characteristics	X	X	X
Genitourinary system	X	X	X

Supplementary Figure S1. 95% Confidence Intervals (Using Z = 1.96) – Updated Data

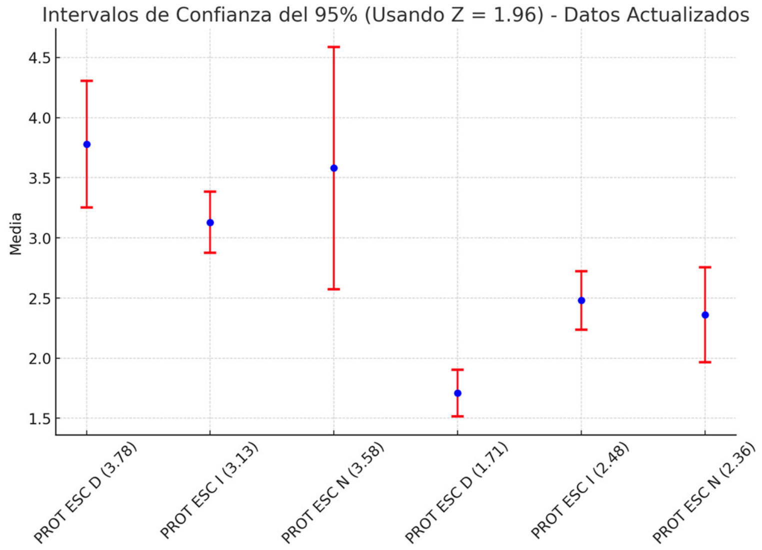

Supplement: Supplementary file 1 [file jcm-14-03870-s001.zip › jcm-3570390-supplementary.pdf]
